# Construction and Characterization of T7 Bacteriophages Harboring Apidaecin-Derived Sequences

**DOI:** 10.3390/cimb44060174

**Published:** 2022-06-01

**Authors:** Tobias Ludwig, Ralf Hoffmann, Andor Krizsan

**Affiliations:** 1 Institute of Bioanalytical Chemistry, Faculty of Chemistry and Mineralogy, Universität Leipzig, Deutscher Platz 5, 04103 Leipzig, Germany; tobias.ludwig@uni-leipzig.de (T.L.); bioanaly@rz.uni-leipzig.de (R.H.); 2 Center for Biotechnology and Biomedicine (BBZ), Universität Leipzig, Deutscher Platz 5, 04103 Leipzig, Germany

**Keywords:** proline-rich antimicrobial peptides, phages, synergy, genetic engineering

## Abstract

The global spread of multi- and pan-resistant bacteria has triggered research to identify novel strategies to fight these pathogens, such as antimicrobial peptides and, more recently, bacteriophages. In a proof-of-concept study, we have genetically modified lytic T7Select phages targeting *Escherichia coli* Rosetta by integrating DNA sequences derived from the proline-rich antimicrobial peptide, apidaecin. This allowed testing of our hypothesis that apidaecins and bacteriophages can synergistically act on phage-sensitive and phage-resistant *E. coli* cells and overcome the excessive cost of peptide drugs by using infected cells to express apidaecins before cell lysis. Indeed, the addition of the highly active synthetic apidaecin analogs, Api802 and Api806, to T7Select phage-infected *E. coli* Rosetta cultures prevented or delayed the growth of potentially phage-resistant *E. coli* Rosetta strains. However, high concentrations of Api802 also reduced the T7Select phage fitness. Additionally, plasmids encoding Api802, Api806, and Api810 sequences transformed into *E. coli* Rosetta allowed the production of satisfactory peptide quantities. When these sequences were integrated into the T7Select phage genome carrying an N-terminal green fluorescent protein (GFP-) tag to monitor the expression in infected *E. coli* Rosetta cells, the GFP–apidaecin analogs were produced in reasonable quantities. However, when Api802, Api806 and Api810 sequences were integrated into the T7Select phage genome, expression was below detection limits and an effect on the growth of potentially phage-resistant *E. coli* Rosetta strains was not observed for Api802 and Api806. In conclusion, we were able to show that apidaecins can be integrated into the T7Select phage genome to induce their expression in host cells, but further research is required to optimize the engineered T7Select phages for higher expression levels of apidaecins to achieve the expected synergistic effects that were visible when the T7Select phages and synthetic Api802 and Api806 were added to *E. coli* Rosetta cultures.

## 1. Introduction

The French-Canadian microbiologist, Felix d’Hérelle, discovered bacteriophages more than 100 years ago [1], but initial interest rapidly faded with the commercialization of synthetic antibiotics in the early 1940s [2]. The rapid global rise of infections with resistant and later multi-resistant bacteria triggered research to identify alternative therapeutics, using novel modes of action, such as antimicrobial peptides (AMPs), and more recently bacteriophages. However, only a few clinical studies on bacteriophage therapies according to modern standards were approved, from 2008 to 2021 there were only 14 clinical trials to treat infectious diseases.

The first clinical study, using phages produced according to GMP standards, was performed from 2015 to 2017. This clinical phase I/IIa study, Phagoburn, aimed at testing a phage cocktail, targeting *Escherichia coli* and *Pseudomonas aeruginosa* infections of burn wounds, for its safety and efficacy. The study ultimately failed because the efficacy was lower than expected, mostly due to an unexpected instability of the phage mixtures prepared months before their application. This showed that further criteria have to be considered for the manufacture of phages and their clinical testing [3]. In one of the most recently completed phase I studies ascending doses of a topically applied *S. aureus* phage preparation were tested [4]. This study was already completed in 2016, but continued with two individual expanded access patient studies, using the *S. aureus* phage preparation and a phage preparation directed against *Pseudomonas aeruginosa* in 2018 [5,6].

**Figure 1:**
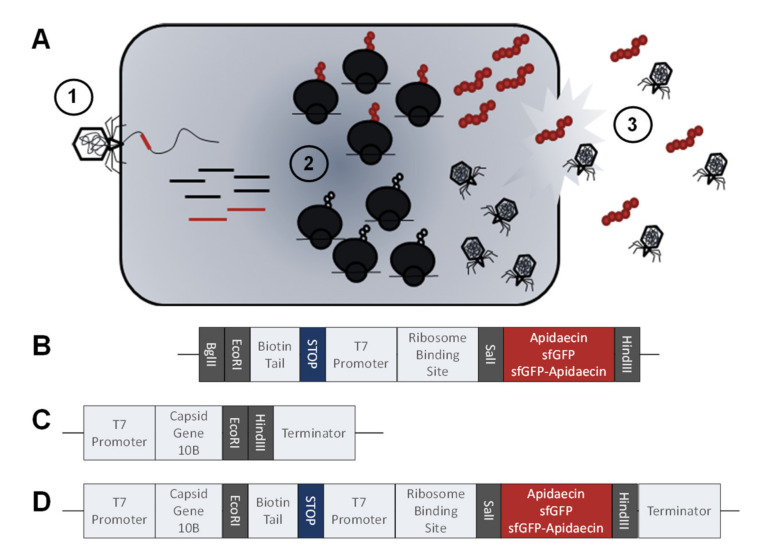
(**A**) Schematic illustration of an *E. coli* cell infected by an engineered phage also encoding an AMP. ① Injection of the phage genome; ② Phage replication and induction of AMP production; ③ Spread of phages and AMP by bacteria lyses; (**B**) DNA fragment encoding for apidaecin, sfGFP, or sfGFP-apidaecin fusion protein; and (**C**) genome of T7Select^TM^ 415 showing region of 10B capsid protein and cloning site; (**D**) Both constructs were used for cloning and integration in the genome of an engineered bacteriophage triggering the expression for apidaecin, sfGFP, or sfGFP-apidaecin fusion protein in the infected *E. coli* cell.

Besides exclusive phage treatments, studies have also evaluated the synergistic effects of phages and antibiotics. Animal studies suggest that the combination of the two compounds drastically reduces the bacterial loads compared to single substance treatments [7,8,9]. Another promising alternative to antibiotics are antimicrobial peptides, which were investigated only in a few pilot studies also indicating synergistic effects between peptides and phages [10,11]. Despite the promising mode of action, phage therapies are limited by a narrow host range and the frequent emergence of phage-resistant bacterial strains during phage therapy, which represent particular concerns in clinical settings [12]. Thus, various approaches were tested to broaden the bacterial host range bottleneck or to change the bacterial host specificity, mainly focusing on the exchange of one or multiple receptor binding proteins (RBPs) [13,14,15,16]. An alternative promising avenue to improve phage effectivity appears to be the integration of genes encoding antimicrobial peptides (AMPs) or proteins into the phage genome to trigger the expression of AMPs by the bacterial host in an early phase of the infectious cycle (Figure 1A). Proof-of-concept studies have already successfully integrated dispersin B, an anti-biofilm protein [17], antimicrobial peptide 1018 [18], and a leaderless bacteriocin targeting Gram-positive bacteria [19] into T7 phages. All of the studies mentioned here integrated the additional sequences downstream of gene product 10, which encodes for the viral capsid. This positioning inside the viral genome is crucial since gp10 marks the beginning of the late stage genes, which are expressed after the viral DNA polymerase is already assembled [19].

However, the significant expression rates of these peptides and proteins only slightly improved the overall activity of the engineered phages. Still, this approach appears helpful to alleviate host range limitations by local production of peptide antibiotics at the site of infection, especially to address phage-resistant bacterial subpopulations. Furthermore, engineered phages might enable treatment of frequently observed poly-bacterial infections [20].

Here we report the construction and characterization of T7 bacteriophages engineered to express apidaecin-related peptides, Superfolder green fluorescent protein (sfGFP), and fusion constructs of sfGFP and apidaecins (Figure 1). Apidaecins are short proline-rich antimicrobial peptides (PrAMPs), derived from the honeybee *Apis mellifera* [21]. This study relied on our previously reported apidaecins Api801 and Api805 [22], as well as analogs Api802 and Api806 derived from these apidaecins (Table 1), respectively, designed to allow a favorable expression in *E. coli*. We demonstrate the synergistic effect of apidaecins and phages, and methods to detect the proteins expressed from sequences integrated into a phage genome. Sequences were integrated downstream of gp10, as depicted in Figure 1D. Although the phage-induced production of peptides and proteins was successful and yielded moderate quantities, the expression rates were too low for a reasonable synergistic effect.

## 2. Materials and Methods

### 2.1. Materials and Chemicals

The reagents, the *E. coli* and phage strains, primer, and plasmids that were used are listed in Table S1 of the Supplementary Materials. The peptides used are listed in Table 1. Water was purified on a Purelab Ultra water purification system (electrical resistivity >18.2 kΩ·m; organic content <2 ppb; ELGA LabWater, Celle, Germany).

### 2.2. Peptide Synthesis

Peptides were synthesized on a multiple synthesizer (SYRO2000, MultiSynTech GmbH, Witten, Germany), using Fmoc/^t^Bu protection strategy, in situ activation with DIC in the presence of HOBt, and Wang resins to yield the peptides as C-terminal acids. Peptides were cleaved with TFA containing 12.5% (*v*/*v*) of a scavenger mixture (ethanedithiol, m-cresol, thioanisole, and water; 1:2:2:2 (by vol)), precipitated with cold diethyl ether, and purified by RP-HPLC on a Jupiter C_18_-column (21.2 mm ID), using an aqueous acetonitrile gradient in the presence of 0.1% TFA. Peptide purities were judged by RP-HPLC on a Jupiter C_18_-column (4.6 mm or 2 mm ID). Monoisotopic masses were confirmed by matrix-assisted laser desorption ionization–time-of-flight mass spectrometry (MALDI-TOF-MS; 5800 Proteomic Analyzer; AB Sciex, Darmstadt, Germany).

### 2.3. Antibacterial Activity

Minimal inhibitory concentrations (MICs) were determined at least twice in triplicates, using a liquid broth microdilution assay in sterile 96-well plates and a total volume of 100 μL per well. Aqueous peptide solutions were serially two-fold diluted in 50 µL of 25% MHBII or LB medium (Luria/Miller). Overnight cultures were diluted in 25% MHBII or LB medium (Luria/Miller) to 1.5 × 10^7^ cells per mL and an aliquot of 50 μL was added to each well. The final cell count in each well was 7.5 × 10^6^ CFU/mL. The plates were incubated for 20 ± 2 h at 37 °C. The turbidity of each well was measured at 600 nm. The MIC was defined as the lowest peptide concentration where the turbidity value did not exceed that of the medium only.

### 2.4. Phage-Peptide-Synergy Assay

*Escherichia coli* Rosetta pLysS was grown in LB medium (Luria/Miller) until an optical density at 600 nm (OD_600_) of ~1 was reached. Bacterial cultures (130 µL) were transferred to a 96-well plate. PrAMPs (10 µL) were added to obtain a final concentration of 128 µg/mL and serially diluted down to 8 µg/mL. A phage solution (10 µL) was added to obtain a final multiplicity of infection (MOI) of 0.0625 and a final volume of 150 µL per well. For the controls, 10 µL of LB medium (Luria/Miller) was added instead of PrAMP and/or phage. OD_600_ were recorded in all wells every 5 min after 20 s of medium orbital shaking at a constant temperature of 37°C for a total of 48 h on a PARADIGM^TM^ (Beckman Coulter, Salzburg, Austria). The GFP fluorescence was recorded (λ_exc_ = 485 nm, λ_em_ = 535 nm) in a second identical plate, which was prepared in parallel using the same conditions, on a GEMINI^TM^ instrument (Molecular Devices, San Jose, CA, USA) every 5 min for 48 h.

### 2.5. DNA constructs for Expression and Genetic Modification of Phages

The construct design of the DNA sequences (Figure 1C), based on the constructs reported by Chen et al. [23], were ordered from Genscript Biotech B.V (Leiden, The Netherlands). These DNA fragments were cloned with BgIII and HindII into pET28a+ plasmid and transformed into chemically competent *Escherichia coli* DH5α by heat shock. Plasmids were isolated and purified, using the QIAprep^®^ Spin Miniprep Kit (Qiagen, Hilden, Germany). Insertion of the DNA sequences of interest was checked by PCR using pET28 primer (Table S1, Supplementary Materials) and sequencing.

### 2.6. Protein and Peptide Expression

The pET28a+ plasmids with different inserts (Table S1, Supplementary Materials) were transformed into chemically competent *Escherichia coli* Rosetta pLysS by heat shock. Colonies were checked by colony PCR, using a pET28 primer (Table S1, Supplementary Materials). An overnight culture of a positive clone was started by inoculating sterile LB medium (Luria/Miller, 5 mL) with one colony picked from an agar plate. The culture was incubated (37 °C, 230 rpm), and the OD_600_ measured. The overnight culture was diluted in fresh LB medium (Luria/Miller, 45 mL) to achieve an OD_600_ of 0.05 and incubated (37 °C, 200 rpm) to reach an OD_600_ of 0.3 to 0.4. The culture was split into two 15-mL cultures and IPTG (c_final_ = 1 mmol/L) added to one culture. Both cultures were incubated for 3 h (37 °C, 200 rpm) and the OD_600_ measured every hour. Afterwards, the bacteria were harvested, lysed, and the lysate separated by SDS-PAGE to confirm protein and peptide expression.

### 2.7. Genetic Modification of Phages

Genetically modified phages were created, using the T7Select 415 Cloning Kit (Merck Millipore, Burlington, VT, USA), according to the manufacturer’s protocol. DNA sequences to be inserted were amplified by PCR, using pET28a+ plasmids and pET primer (Table S1, Supplementary Materials). The PCR product was digested with EcoRI and HindIII and ligated into the predigested T7Select vector arms (16 h, 4 °C), using T4 ligase. The ligated T7Select genome (Figure 1, panels B and D) was added to the provided packaging extract and incubated at room temperature. After 2 h, a plaque assay was used to obtain infectious particles. After 18 ± 2 h, plaques were cut out, transferred into LB medium (Luria/Miller, 450 µL) and incubated (37 °C). After 2 h, chloroform (30 µL) was added, and the correct insertion verified by PCR, using T7 primer (Table S1, Supplementary Materials) and sequencing.

### 2.8. Plaque Assay

*E. coli* Rosetta pLysS was grown until an OD_600_ of ~1. phage lysates were prepared in a serial dilution from 10^−1^ to 10^−9^. Aliquots of the *E. coli* culture (250 µL) were transferred to molten LB (Luria/Miller) top agar (0.75% agar; 4 mL), the diluted phage lysates were added (100 µL), and the mixture was poured onto solid LB (Luria/Miller) agar plates (1.5% agar). After solidification of the top agar, the plates were turned upside down and incubated at 37 °C overnight. The plaques were counted, and the phage titers were calculated by multiplying plaque counts and dilution factors.

### 2.9. Time Kill Assay

*E. coli* Rosetta pLysS was grown until OD_600_ of ~1 and aliquoted on a clear 96-well plate (90 µL/well) before 10 µL phage lysate was added to obtain a MOI of 0.0625. The outer rim of the 96-well plate, i.e., rows A and H and columns 1 and 12, were filled with water (100 µL/well) to reduce edging effects. The plate was incubated at 37 °C for at least 48 h and the OD_600_ was recorded every 15 min with 5 s medium strength orbital shaking prior to each measurement.

### 2.10. Mixed Culture Liquid Assay

A fresh *E. coli* Rosetta pLysS culture was grown. Upon reaching an OD_600_ of 0.4, phages were added (MOI of 0.01) and the culture incubated (37 °C, 400 rpm). After 30 min, aliquots (50 µL) of each phage were transferred to ten wells of a 96-well plate. In parallel, a phage-resistant *E. coli* Rosetta strain (R2.3), isolated from a plaque assay, was grown until an OD_600_ of ~0.3 and diluted with medium to an OD_600_ of 0.04. Prior to adding this resistant culture to the 96-well plate containing the phage-sensitive bacteria and a phage, IPTG was added to the resistant culture (final concentration of 0.1 mmol/L) to induce expression of GFP. The phage-insensitive culture was added (50 µL) and incubated at 37 °C. The fluorescence of GFP (λ_exc_ = 485 nm, λ_em_ = 535 nm) was measured, using a PARADIGM^TM^ microplate reader (Beckman Coulter) every 15 min for 12 h.

## 3. Results

### 3.1. Activity of Apidaecin Analogs against E. coli

**Figure 2:**
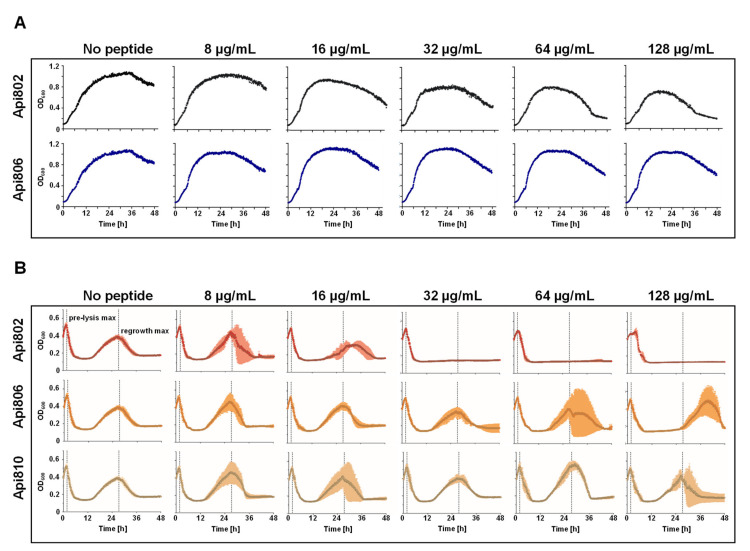
(**A**) Influence of apidaecin analogs Api802 (black) and Api806 (blue) added in increasing concentrations to an *E. coli* Rosetta pLysS culture in LB medium and incubated at 37 °C; (**B**) Influence of the studied apidaecin analogs on phage-mediated lysis including the appearance of potentially phage-resistant bacteria. Api802 (red), Api806 (orange), and Api810 (beige) were added in increasing concentrations to an *E. coli* Rosetta pLysS culture in LB medium, inoculated with T7Select phage (MOI 0.0625), and incubated at 37 °C. The OD_600_ was recorded every 5 min for 48 h. Dashed lines indicate the maximal OD_600_ values before lysis (pre-lysis max) and the highest OD_600_ values of the regrowth phase (regrowth max) of a control experiment without addition of a peptide. Experiments were completed twice in triplicates. Error bars show the standard deviation of all three replicates of one representative experiment.

The previously reported lead structures, Api801 and Api805 [22], were substituted at the N-terminus, i.e., Gly1Met, to obtain higher expression rates of Api802 and Api806, respectively, in *E. coli* (Table 1). The MICs of Api802 (2–4 µg/mL) and Api806 (4–8 µg/mL) were comparable to Api801 (2 µg/mL) and Api805 (4 µg/mL), respectively, in *E. coli* Rosetta DE3 pLysS grown in 25% MHBII (Table 1). In LB medium, which was used for all of the phage experiments, the MICs of Api802 and Api806 increased to 64 µg/mL and 128 µg/mL, respectively. When added to a growing *E. coli* Rosetta culture, Api802 reduced the growth-rate at 32 µg/mL, which increased further at 64 and 128 µg/mL (Figure 2A). Api806 showed no significant effect on the growing culture at all of the tested concentrations (Figure 2A). Additionally, Api810 was synthesized as a negative control by substituting Arg-17 in Api801 by alanine [24,25], which indeed was inactive in both media (MIC ≥ 128 µg/mL).

### 3.2. Api802 Affects Phage Mediated Lysis

As the phage to be engineered should express Api802 or Api806 simultaneously with the other phage-encoded proteins, the effect of both AMPs added to an *E. coli* culture infected with a T7-phage was investigated first. Since apidaecin and its analogs inhibit protein translation by targeting the 70S ribosome [26], we expected an effect on phage production. This effect could reduce phage-mediated lysis but might also reduce the emergence and growth of phage-resistant bacteria, which frequently happens after the initial lysis phase [27,28,29]. Phage-mediated lysis started 2 h after phage addition and regrowth of an *E. coli* Rosetta DE3 pLysS population, which was potentially phage-resistant, was observed with a maximal growth 24 h after the initial lysis in growth experiments with T7select phage without the addition of apidaecin (Figure 2B). Therefore, Api802, Api806, and the control peptide Api810 were added to *E. coli* Rosetta DE3 pLysS cultures incubated with the T7Select phage and the growth of the culture was regularly monitored (Figure 2B). Only the highest concentration of Api802 corresponding to 2x MIC, i.e., 128 µg/mL, showed a significant effect on the lysis kinetics of the T7Select phage, delaying the pre-lysis phase compared to a control experiment without addition of a peptide by ~2.5 h considering the maximal OD_600_ value of the cell culture. Api806 and Api810 did not delay the pre-lysis phase. The regrowth was already delayed for an Api802 concentration of only 16 µg/mL, i.e., 0.25× MIC in LB medium, and completely inhibited at higher peptide concentrations for the full 48 h observation period. Api806 delayed regrowth only at 128 µg/mL, which corresponds to its MIC in LB medium, while Api810 had no visible effect. Thus, Api802 appeared to be a promising candidate for phage engineering, since it did not reduce the lytic activity of the phage and prevented growth of the potentially phage-resistant bacteria in a small concentration range.

### 3.3. Generation of sfGFP Reporter Phages

As proof of concept for proper phage integration and phage-mediated protein expression, three plasmids containing sfGFP, sfGFP-Api801, or sfGFP-Api805 were constructed and integrated in *E. coli* Rosetta pLysS. When sfGFP expression was induced in the corresponding *E. coli* strain, the measured fluorescence of sfGFP and OD_600_ values indicated high protein quantities without reducing bacterial growth, while expression of both of the sfGFP apidaecin fusion constructs reduced the growth rate, with sfGFP-Api801 almost stopping bacterial growth (Figure S1A). Since both of the apidaecin sequences were added to the C-terminal sequence of sfGFP, leaving the active C-terminus of the apidaecins unaltered, we switched the order of sfGFP-Api801, elongating the sequence of sfGFP at the N-terminus with a peptide sequence (Api802-sfGFP). However, the fluorescence and OD_600_ values measured for Api802-sfGFP were identical to sfGFP-Api801. Importantly, the expression of sfGFP and sfGFP-apidaecin constructs deduced from the fluorescence were confirmed by the band intensities obtained in SDS-PAGE (Figure S1B).

**Figure 3:**
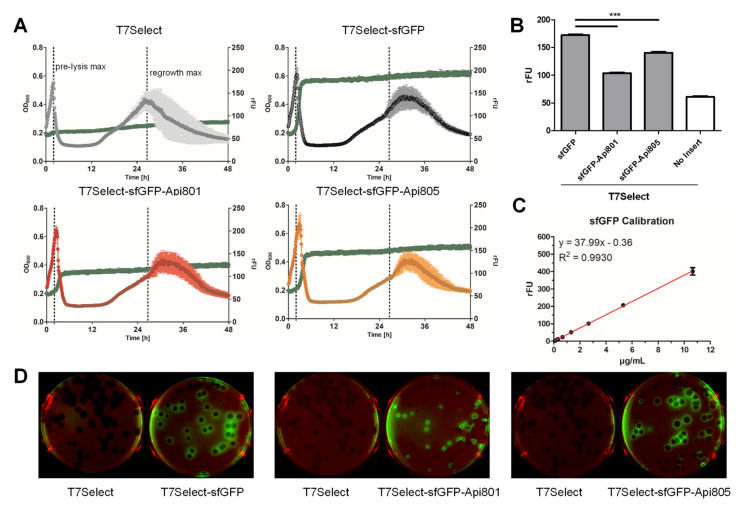
Expression of sfGFP and sfGFP fused to either Api801 or Api805 monitored by the fluorescence of GFP. (**A**) OD_600_ and fluorescence (green curve) of *E. coli* Rosetta cultures inoculated with T7Select (gray), T7Select-sfGFP (black), T7Select-sfGFP-Api801 (red), or T7Select-sfGFP-Api805 (orange) phages (MOI 0.0625) were monitored every 5 min for 48 h. Dashed lines indicate the maximal OD_600_ values before lysis (pre-lysis max) and the highest OD_600_ values of the regrowth phase (regrowth max) of T7Select; (**B**) Upper fluorescence plateau obtained for the tested phages. *** *p* < 0.0001 for T7Select-sfGFP versus T7Select-sfGFP-Api801 or T7Select-sfGFP-Api805. (**A**,**B**) Experiments were completed twice in quintuplicates. Error bars show the standard deviation of all five replicates of one representative experiment; (**C**) Serial dilution of recombinant sfGFP used to quantify the expressed sfGFP constructs; (**D**) Phage-mediated GFP expression was tested in a plaque assay.

Confirming the successful plasmid-based expression of sfGFP and sfGFP-Api801/Api805 constructs in *E. coli*, the coding DNA segments were integrated into the T7Select phage. Bacterial lysis patterns of all three phages were comparable to the parent T7Select phage, but the regrowth phase was shifted for ~4.8 h for all three sfGFP-phages (Figure 3A). The highest fluorescence was observed for T7Select-sfGFP, which significantly decreased for T7Select-sfGFP-Api805, and even further for T7Select-sfGFP-Api801 (Figure 3B). A plaque assay performed with the engineered phages confirmed the bacterial expression of sfGFP and both sfGFP apidaecin constructs, as indicated by the fluorescence localized at the outer rim of the plaques (Figure 3D). Sequencing of the inserts confirmed the proper integration. Based on a sfGFP calibration curve (Figure 3C), the protein quantities expressed in the cell culture were ~2.9 µg/mL of sfGFP, 1.13 µg/mL of sfGFP-Api801, and 2.10 µg/mL of sfGFP-Api805, which translate to 82,010, 27,675, and 51,040 copies per cell, respectively. Thus, the C-terminal Api801 and Api805 sequences reduced sfGFP expression to around one-third and two-thirds, respectively. Assuming a burst size of 180 T7Select phages per *E. coli* cell [30] and a copy number of 415 proteins per phage [31], these numbers are close to the theoretically expected yield of 74,700 molecules per cell. Thus, sfGFP was expressed at the normal cellular capacity. Considering these numbers and assuming that the proteins are uniformly distributed in *E. coli* with an average cell volume of ~4 fL [32], the intracellular concentrations would be ~109 nmol/L for sfGFP, 37 nmol/L for sfGFP-Api801, and ~68 nmol/L for sfGFP-Api805. Assuming that peptides Api801/2 and Api805/6 would be expressed at the same level as the corresponding sfGFP constructs, the intracellular concentrations would be ~0.13 µg/mL and ~0.27 µg/mL, respectively.

### 3.4. Api802 Delays Bacterial Protein Expression of Phage Proteins

**Figure 4:**
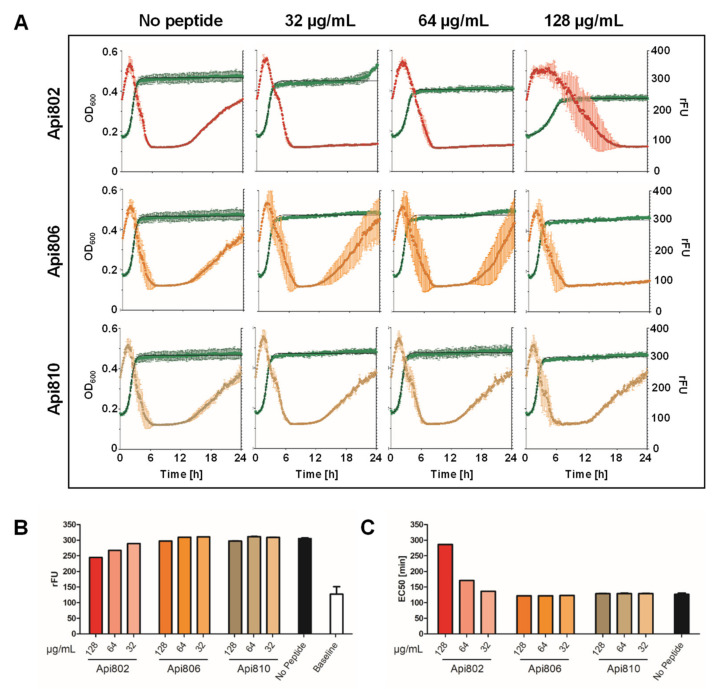
Influence of Api802, Api806, and Api810 on phage-mediated expression of sfGFP in *E. coli* Rosetta pLysS infected with T7Select-sfGFP phage (MOI 0.0625). (**A**) OD_600_ and relative sfGFP fluorescence intensities (green curve) of *E. coli* cultures incubated with Api802 (red), Api806 (orange), or Api810 (beige); (**B**) Maximal relative fluorescence intensities (rFU) and (**C**) EC_50_ values observed for the datasets shown in panel A. (**A**–**C**) Experiments were completed twice in triplicates. Error bars show the standard deviation of all three replicates of one representative experiment.

To investigate if the lysis delaying effect of Api802 added to the culture medium (Figure 2B) relies on the inhibition of protein synthesis, the phage-peptide-synergy assay was repeated with the T7Select-sfGFP-phage for different peptide concentrations monitoring both OD_600_ and fluorescence (Figure 4A). When Api802 was added at the highest concentration of 128 µg/mL, the fluorescence in the cell culture was reduced from 305 rFU in the absence of a peptide to 247 rFU. After subtraction of the baseline level (no sfGFP phage), this accounted for a reduction of 31.5%, compared to a reduction of ~20.7% for Api802 at a concentration of 64 µg/mL. Api806 and Api810 affected sfGFP expression only slightly at the highest concentration.

A stronger effect was observed for the time to reach the half-maximal fluorescence intensity (EC_50_) indicating the expression rate of sfGFP (Figure 4C). At the highest concentration, the EC_50_ value of Api802 was 286 min compared to 127 min for the control (no peptide), which is an increase of more than 125%. At a concentration of 64 µg/mL the EC_50_ still increased by 44 min or 34.6%, while the EC_50_ values of Api806 and Api810 were similar to the control at all concentrations. This supports the hypothesis that the lysis is delayed by Api802 due to inhibition of phage protein production by blocking the 70S ribosome, which supports the notion that Api802 might be a suitable candidate only when present at lower concentrations. Api806 might still be a promising candidate, as it does not show any adverse effects on phage fitness.

### 3.5. Generation and Activity of PrAMP-Phages

The data suggested that DNA sequences can be successfully integrated in the phage genome to express the corresponding proteins at reasonable quantities in infected *E. coli* cells. Thus, we tested if *E. coli* can also express the peptides Api802, Api806, and Api810 in sufficient amounts when the corresponding plasmids were transformed into *E. coli* Rosetta pLysS. In the presence of IPTG, all of the *E. coli* strains harboring the plasmids with an apidaecin analog or with an empty pET28a+ vector, reduced the growth compared to the uninduced controls (Figure S2A), which was most pronounced for Api802. Three hours after IPTG induction, the cells were harvested, lysed, and the protein preparation loaded on SDS-PAGE (Figure S2B). The Coomassie stain displayed strong bands for Api806 and Api810 in the expected region, based on the migration of the pure peptides. Both peptides were confirmed by mass spectrometry after in-gel digestion with trypsin. The Api802 band was not visible in the gel, but mass spectrometry confirmed its presence in the expected region.

**Figure 5:**
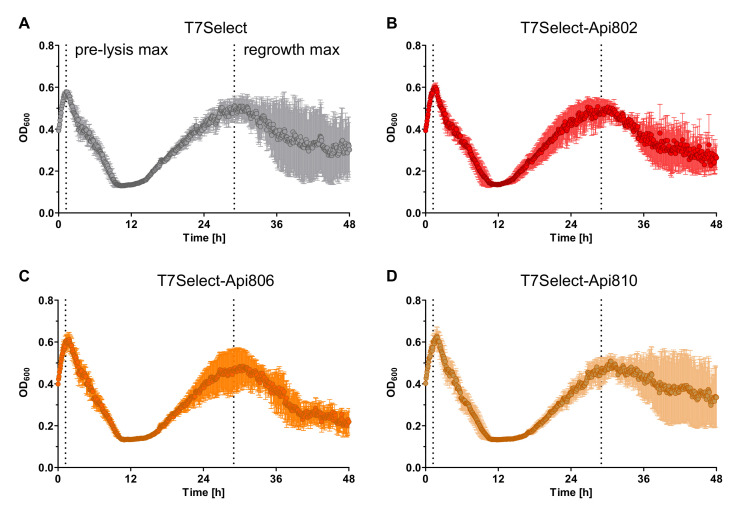
Bacterial growth of *E. coli* Rosetta cultures infected with engineered T7Select phages (MOI 0.0625) without (**A**) (gray) or with Api802 (**B**) (red), Api806 (**C**) (orange), or Api810 (**D**) (beige) inserts. Shown are the OD_600_ values recorded every 5 min for 48 h. Dashed lines indicate the highest optical density before lysis (pre-lysis max) and the highest optical density of regrowth phase (regrowth max). Experiments were completed twice in quintuplicates. Error bars show the standard deviation of all five replicates of one representative experiment.

Next the T7Select phages containing the sequences of Api802, Api806, and Api810 were engineered. All phages produced infectious particles, as confirmed by their activity in time kill assays (Figure 5A–D) and the subsequent determination of increased PFU/mL. All phages showed a similar lysis behavior, and afterwards the cell cultures showed a significant growth of potentially phage-resistant bacteria. A similar phage proliferation was observed for the T7Select, T7Select-Api802, T7Select-Api806, and T7Select-Api810 with 6.3 × 10^8^ PFU/mL, 5.3 × 10^8^ PFU/mL, 6 × 10^8^ PFU/mL, and 8.7 × 10^8^ PFU/mL, respectively. The peptide sequences integrated into the phage genome did not affect phage proliferation.

### 3.6. Mixed Culture Liquid Assay with PrAMP-Phages

The envisioned application of engineered phages initiating the expression of antimicrobial peptides would be an additional antimicrobial effect on phage-resistant bacteria growing in close vicinity to the phage host. Thus, an *E. coli* Rosetta strain carrying a GFP plasmid was trained to be resistant to the T7Select phage by picking single colonies from an agar plate after streaking phage-infected *E. coli* cultures on the plate (Figure S3). The growth rate of this phage-resistant *E. coli* R2.3 strain was neither affected by the T7Select phage nor any of the three engineered T7Select apidaecin-coding phages (Figure S3). Interestingly, the *E. coli* R2.3 was slightly more susceptible to Api802 (MIC = 2 µg/mL) and Api806 (MIC = 4 µg/mL) than against the original *E. coli* Rosetta.

**Figure 6:**
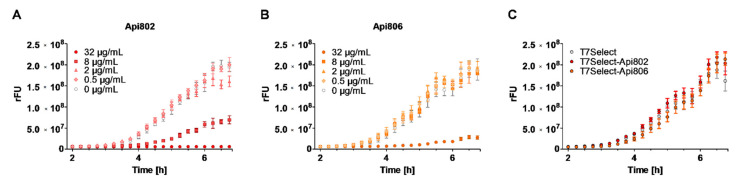
Expression of GFP by *E. coli* R2.3 monitored by its inherent fluorescence in the presence of different Api802 (**A**) and Api806 (**B**) concentrations and engineered apidaecin phages (**C**). (**A**,**B**) *E. coli* Rosetta was pre-incubated with T7Select (MOI 0.01) in LB medium for 30 min, before different concentrations of Api802 (**A**) and Api806 (**B**) as well as an *E. coli* R2.3 culture (10-fold lower cell count) were added. GFP expression was induced with IPTG; (**C**) *E. coli* Rosetta was pre-incubated with T7Select, T7Select-Api802, or T7Select-Api806 (MOI 0.01) in LB medium. *E. coli* Rosetta R2.3 was added in 10-fold lower cell numbers. GFP expression was initiated with IPTG. Experiments were completed twice in ten replicates. Error bars show the standard deviation of all ten replicates of one representative experiment.

*E. coli* Rosetta was preincubated with the T7Select, T7Select-Api802, or T7Select-Api806 phages for 30 min before the phage-resistant *E. coli* R2.3 was added in the presence of IPTG to induce the expression of GFP. This should allow quantitation of the expression rate of GFP by its inherent fluorescence in the phage-resistant *E. coli* strain indicating the inhibition of protein expression by an apidaecin analog released from a nearby phage-infected and burst *E. coli* Rosetta cell. This experimental setup was tested by adding different quantities of Api802 or Api806 to a culture of *E. coli* Rosetta infected with the T7Select phage after addition of *E. coli* R2.3. Api802 reduced the GFP expression when added at concentrations of 2 µg/mL or more, and completely inhibited GFP expression at 32 µg/mL (Figure 6A). Expectedly, Api806 showed a weaker effect with a strong inhibition observed at 32 µg/mL (Figure 6B). However, when the experiment was performed without addition of peptides but with Api802- and Api806-coding phages, the fluorescence intensities in all of the cell cultures increased constantly with the same slope reaching very similar intensities after 6 h. Compared to the T7Select phage, the engineered phages had no measurable effect on GFP expression indicating that the medium concentrations of presumably released Api802 and Api806 were insufficient for affecting the protein expression in *E. coli* R2.3 cells (Figure 6C).

## 4. Discussion

Even though phages are re-emerging as potential alternatives to common antibiotics, therapeutic applications are limited due to considerable pharmacological disadvantages. First, many phages possess a narrow host range, typically limited to a certain bacterial strain [33]. Although this is not a shortcoming per se, it limits the use of phages as broad-spectrum antimicrobial agents. Second, even susceptible bacteria can develop phage-resistance relatively fast, already during the first treatment, which further complicates the clinical applications [34]. Based on these considerations, we created genetically engineered lytic phages to target phage-sensitive bacteria and to induce the expression of PrAMPs in the host, which attack nearby phage-resistant bacteria after being released from the lysed host cell.

In our own experiments, we regularly observed the development and growth of potentially phage-resistant bacteria. Similar to recent studies suggesting synergistic action between phages and conventional antibiotics [9], we observed dose-dependent synergistic effects between apidaecin-derived PrAMPs and phages. The apidaecin-derived peptides suppressed the growth of potentially phage-resistant bacteria in *E. coli* cultures inoculated with the unmodified T7Select phage. As these peptides are known to inhibit the 70S ribosome and thus reduce protein synthesis, they should also suppress phage production in the host cells [22,26,35]. Thus, we expected a strong influence on phage-induced cell lysis, but it occurred only at high concentrations of Api802, while the suppressing effect on the growth of potentially phage-resistant bacteria dominated at 32 µg/mL. Api806 delayed this growth only slightly at the highest tested concentration of 128 mg/mL (Figure 2B). Accordingly, concentrations of Api802 from 32 to 128 µg/mL delayed the increase in fluorescence and reduced maximum fluorescence in the cell cultures infected with the sfGFP reporter phage, whereas Api806 had no influence (Figure 4). This suggests that high concentrations of Api802 probably reduce the phage fitness. Thus, the concentration of administered PrAMPs must be carefully balanced so that it does not slow down production of phage progeny.

The successful genomic integration of peptide and protein sequences was already demonstrated for the phage display kit T7Select 415-1b [17,18], including the expression of detectable quantities of the fluorescent protein mCherry [18]. Here we demonstrate that T7Select can also produce sfGFP and sfGFP-PrAMP fusion proteins in both liquid culture (Figure 3A) and solid agar (plaque assay, Figure 3D) that can be monitored by the fluorescence of GFP. However, the protein yields of the sfGFP-PrAMP constructs were substantially lower than the yields of sfGFP, due to the inhibitory effects of PrAMPs on the 70S ribosome. Considering the fluorescence data and assuming an equal expression of Api802 and Api806, the intracellular peptide concentrations would be ~0.13 µg/mL and ~0.27 µg/mL, respectively. The fact that Api802, Api806, and Api810 were not detected in the lysates of lysed bacterial cells infected by the T7Select-Api802, Api806, or Api810 phages by SDS PAGE or LC-MS (data not shown) suggests that the peptide concentrations might be lower. Although less likely, it cannot be excluded that the peptides stick to other cellular compartments and were thus lost during sample preparation. The low concentrations are consistent with the findings of Lemon et al., who were also unable to detect phage-produced peptide 1018 in LC-MS, providing a limit of detection of 1 ng/L [18]. There were other approaches to construct the T7 phages for production of antimicrobial peptides or proteins, but in all cases the production of the integrated peptides or proteins was not confirmed [17,19]. Thus, phage-mediated expression of AMPs appears to happen at levels well below the detection limits of the applied analytical techniques. This also confirms our findings, that PrAMP levels were insufficient for the envisaged antimicrobial activities.

This notion is further supported by the fact that cell lysis initiated by the engineered T7Select-Api802 and T7Select-Api806 phages and phage propagation was similar to the original T7Select phage. Due to the low concentrations of expressed PrAMPs by phage induction, no adverse effect was observed on phage fitness. Also, a similar growth phase of potentially phage-resistant bacteria was observed indicating that the PrAMPs integrated in the phage genome were expressed in low quantities. Similarly, both of the PrAMP-phages did not reduce sfGFP expression in the co-cultured phage-resistant strain *E. coli* R2.3, as the produced peptide concentrations were certainly well below the peptide concentrations in the control experiments, i.e., 8 µg/mL for Api802 and 32 µg/mL for Api806.

We would like to stress that all phage-related experiments were performed in LB medium, which is not usually the optimal choice for physiological studies. It provides conditions for good bacterial growth and peptide expression, but reduces the activity of PrAMPs at least 30-fold compared to standard 25% MHBII, which is primarily used to determine MICs of antimicrobial agents and still provides an ideal medium for bacterial growth. Thus, we tested the T7Select phages in 25% MHBII medium, but the bacterial growth and protein expression rates were reduced, which also affected phage lysis (Figure S4A). Compared to LB medium, a lower and delayed pre-lysis maximum was observed and, interestingly, no bacterial regrowth happened. Therefore, investigation of the growth-suppressing effect on the potentially phage-resistant bacteria of synthetic apidaecin or apidaecin expressing phages would be impossible. Additionally, expression rates of GFP were drastically reduced when *E. coli* R2.3 was co-cultured with *E. coli* Rosetta pLyS in 25% MHBII and again, the DNA insertions encoding for apidaecins into the T7Select phage had no visible effect (Figure S4B). Thus, *E. coli* cultured in 25% MHBII are more susceptible to PrAMPs, but this medium further reduces the already low expression levels in cells infected with apidaecin phages. Hence, further research is required to find the optimized conditions balancing high peptide susceptibility, high lytic rates, and high expression levels. Additionally, the phage-mediated expression could be enhanced by insertion of multiple copies of a peptide sequence. Another approach could be attaching peptides via a cleavable linker to the viral capsid. Another promising but more challenging strategy would be the addition of secretion signals to the peptide sequence to prevent their intracellular accumulation with adverse effects on the host cell and thus phage replication and peptide expression by secreting the peptides to the periplasm or the medium.

## 5. Conclusions

Phages appear to be promising alternatives to antibiotic therapies, but maybe even more important could be the combination with antibiotics or antimicrobial peptides, as studied here for PrAMPs showing a synergistic effect by suppressing the rapid occurrence of phage-resistant strains. Engineered phages expressed the desired GFP-PrAMPs at moderate levels, which provides a valid base for further improvements of the antibacterial activity of the phages. However, these modifications have to be well-balanced and carefully performed to improve phage efficiency. Further studies are needed to optimize the in vitro conditions and to improve peptide expression.

## Figures and Tables

**Table 1:** Sequences and MICs of antimicrobial peptides included in this study against *E. coli* Rosetta DE3 pLysS cultured in 25% MHBII or LB medium. n.d. = not determined.

PrAMP	Sequence	MIC [µg/mL]	
		25% MHBII	LB Medium
Api801	GNNRPVYIPRPRPPHPRL-OH	2	n.d.
Api802	MNNRPIYIPRPRPPHPRL-OH	2–4	64
Api805	GNNRPIYIPRPRPPHPRPIRV-OH	4	n.d.
Api806	MNNRPIYIPRPRPPHPRPIRV-OH	4–8	128
Api810	MNNRPIYIPRPRPPHPAL-OH	128	>128

## Data Availability

The data presented in this study are available on request from the corresponding author.

## Supplementary Materials

The following supporting information can be downloaded at: https://www.mdpi.com/article/10.3390/cimb44060174/s1, File S1: Material and Chemicals—Reagents, Table S1: Bacterial and phage strains, plasmids, and primers, Figure S1: Expression of sfGFP-apidaecin constructs and growth-related effects, Figure S2: Expression of apidaecins and growth-related effects, Figure S3: Isolation and Growth of T7Select phage-resistant *E. coli* Rosetta strain R2.3, Figure S4: Mixed Culture Liquid Assay in 25% MHB and 100% LB medium
