# Supplementary material for: Construction and Characterization of T7 Bacteriophages Harboring Apidaecin-Derived Sequences
Source: Curr Issues Mol Biol. 2022 Jun 1;44(6):2554–68. doi: 10.3390/cimb44060174 (PMC9221748; doi:10.3390/cimb44060174)
Supplement: Supplementary file 1 [file cimb-44-00174-s001.zip › Supplementary material.pdf]

Supplementary material

Construction and characterization of T7 bacteriophages harboring apidaecin-derived sequences

Tobias Ludwig, Ralf Hoffmann, and Andor Krizsan

Institute of Bioanalytical Chemistry, Faculty of Chemistry and Mineralogy, and Center for Biotechnology and Biomedicine, Universität Leipzig, Leipzig, 04103, Germany

Content

File S1: Material and Chemicals - Reagents S2

Table S1: Bacterial and phage strains, plasmids, and primers S3

Figure S1: Expression of sfGFP-apidaecin constructs and growth-related effects S4

Figure S2: Expression of apidaecins and growth-related effects S5

Figure S3: Isolation and Growth of T7Select phage-resistant *E. coli* Rosetta strain R2.3 .... S6

Figure S4: Mixed Culture Liquid Assay in 25% MHB and 100% LB medium S7

File S1: Materials and Chemicals

Reagents were obtained from the following companies unless stated otherwise: AppliChem GmbH (Darmstadt, Germany): Ethidium bromide solution (1%) and tris(hydroxymethyl)aminomethane (Tris); Biosolve BV (Valkenswaard, Netherlands): Acetonitrile (HPLC-S gradient grade) and dimethylformamide (DMF, peptide synthesis grade); Bio-Rad Laboratories GmbH (Munich, Germany): Precision Plus Protein™ Dual Xtra protein standard; Carl Roth GmbH, Karlsruhe, Germany): Agar-Agar Kobe I, IPTG (≥99%), kanamycin, lysogeny broth (LB) Miller, lysozyme (≥45 000 FIP U/mg), magnesium chloride (≥99%), phosphate buffered saline (PBS, pH 7.4), potassium chloride (≥99%), potassium dihydrogen phosphate (≥99%), sodium dodecyl sulfate (SDS, >99.5%), sodium hydroxide (≥98%), and trichloroacetic acid (≥99%); Greiner Bio-One GmbH (Frickenhausen, Germany): 96-Well microtiter plates; Honeywell Fluka™ (Seelze, Germany): Ammonium chloride (≥99.8%), calcium chloride (≥ 99.5 %), and magnesium chloride (≥99%); Iris Biotech (Marktredwitz, Germany): Leucin-Wang resin; Merck KGAa (Darmstadt, Germany): Diethyl ether (puriss); MultiSynTech GmbH: 4-Benzyloxybenzyl alcohol (Wang) resin; Orpegen Pharma GmbH (Heidelberg, Germany) or MultiSynTech GmbH (Witten, Germany) or Iris Biotech: All 9-fluorenylmethoxycarbonyl- (Fmoc) protected amino acids; New England Biolabs (Ipswich, U.S.A.): restriction enzymes EcoRI, BglII, and HindIII and T4 ligase; Phenomenex Inc. (Torrance, CA, USA): Jupiter C_{18}-columns (internal diameter (ID): 21.2 mm, length: 250 mm, particle size: 15 µm, pore size: 30 nm; ID: 10 mm, length: 250 mm, particle size: 5 µm, pore size: 30 nm; ID: 2 mm, length: 150 mm, particle size: 5 µm, pore size: 30 nm); SERVA electrophoresis GmbH (Heidelberg, Germany): Acrylamide/bisacrylamide (30% T, 2.67% C), agarose, ammonium persulfate (99%), Coomassie brilliant blue G250, glycine (98.5-101%), protease inhibitor mix, N,N,N′,N′-tetramethylethylendiamin (TEMED), Tween® 20 (pure), and trypsin (sequencing grade, MS approved); Sigma-Aldrich GmbH (Taufkirchen, Germany): m-Cresol (99%), N,N-diisopropylcarbodiimide (DIC, >98% by GC), disodium hydrogen phosphate × 12 H2O (≥99%), 1,2-ethanedithiole (≥98%), 1-hydroxy-benzotriazole (HOBt, >98%), magnesium sulfate (>97%), 2-mercaptoethanol (≥ 99%), Müller Hinton broth II (MHBII), potassium chloride (>99%), potassium phosphate (≥ 99 %), sodium acetate (>99%), sodium chloride (≥99,5%), thioanisole (≥99%), trifluoroacetic acid (TFA, UV-grade for HPLC), TFA (purum) for peptide synthesis, and tris(hydroxymethyl)aminomethane (tris) acetate EDTA buffer (10x, TAE); Thermo Scientific GmbH (Schwerte, Germany): DNase I (RNase-free, 1 U/µL), dNTP Mix (2 mmol/L each) and Phusion High-Fidelity DNA Polymerase (2U/µL); VWR (Dresden, Germany): Chloroform (≥99%).

Table S1: List of all bacterial and phage strains, plasmids, and primers used in the current study.

	Genotype/Sequence	Reference
**Bacterial Strain**		
E. coli DH5α	F^{-} *endA1 glnV44 thi-1 recA1 relA1 gyrA96 deoR nupG purB20* φ80d*lacZ*ΔM15 Δ(*lacZYA-argF*)U169, hsdR17($r_K^- m_K^+$), λ$^-$	Invitrogen
E. coli Rosetta^{TM} pLysS	F$^-$ *ompT gal dcm lon*? *hsdS*$_B$($r_B^- m_B^-$) λ(DE3) [*malB*$^+$]$_{K\text{-}12}$(λ^S) pLysSRARE(Cm^R)	Merck
E. coli Rosetta R2.3	+ pGFP / T7Select^{TM} 415 resistant	This publication
**Phage Strain**		
T7Select^{TM} 415	T7 Δgp0.3-07, Δgp3.8, gp10B+MCS	Merck
T7Select_sfGFP	+ Biotin s1.3 sfGFP	
T7Select_sfGFP-Api801	+ Biotin s1.3 sfGFP-Api801	
T7Select_sfGFP-Api805	+ Biotin s1.3 sfGFP-Api805	This publication
T7Select_Api802	+ Biotin s1.3 Api802	
T7Select_Api806	+ Biotin s1.3 Api806	
T7Select_Api810	+ Biotin s1.3 Api810	
**Plasmid**		
pGFP	pET Biotin 6xHis GFP Kan^R	Addgene
pET28a$^+$	High copy number, pBR322-derived expression plasmid (5.369 kb), 6xHis-tag and T7 promoter, induction by IPTG, Kan^R	Novagen
psfGFP	pET28a$^+$ Biotin s1.3 sfGFP	
psfGFP-Api801	pET28a$^+$ Biotin s1.3 sfGFP-Api801	
psfGFP-Api805	pET28a$^+$ Biotin s1.3 sfGFP-Api805	
pApi802-sfGFP	pET28a$^+$ Biotin s1.3 Api802-sfGFP	This publication
pApi802	pET28a$^+$ Biotin s1.3 Api802	
pApi806	pET28a$^+$ Biotin s1.3 Api806	
pApi810	pET28a$^+$ Biotin s1.3 Api810	
**Primer**		
pET28 fwd	TTATGCTAGTTATTGCTCAGCGG	
pET28 rev	GGAGCTGTCGTATTCCAGTC	This publication
T7 fwd	GGAGCTGTCGTATTCCAGTC	
T7 rev	AACCCCTCAAGACCCGTTTA	

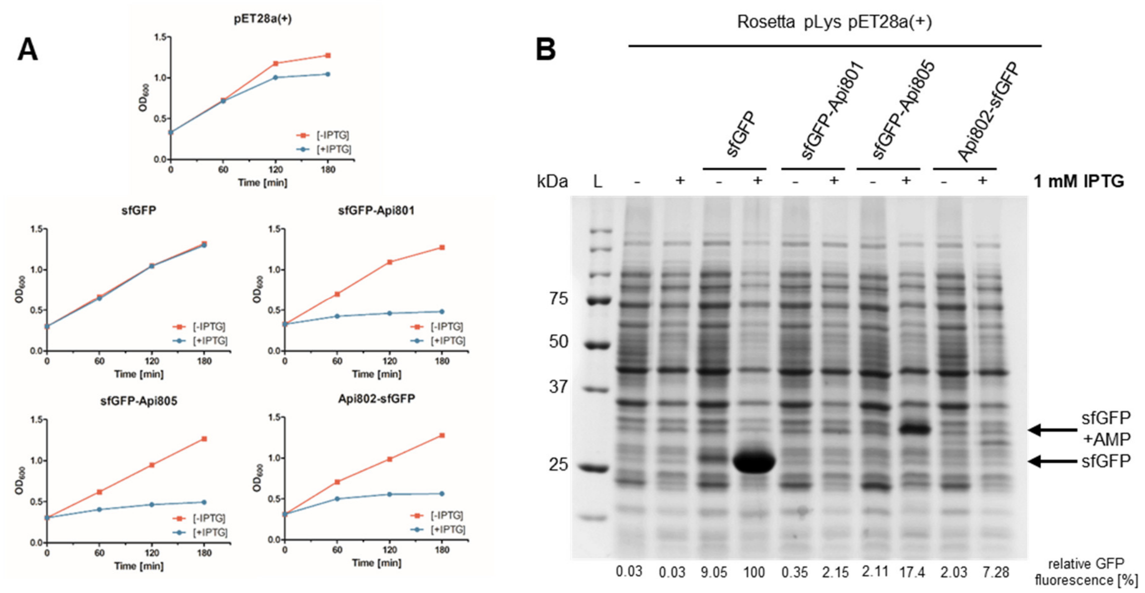

Figure S1. Expression of sfGFP, both sfGFP-Api801/805 constructs, and the Api802-sfGFP construct in *E. coli* Rosetta pLysS. **A** Comparison of growth-related effects after IPTG-induced expression of the indicated constructs using the OD_{600} values of the cell culture. *E. coli* culture growth rates monitored by OD_{600} values without (red) or with (blue) inducing peptide expression by IPTG. **B** SDS-PAGE of the corresponding *E. coli* lysate protein preparations. The relative fluorescence intensities of the corresponding *E. coli* cultures are indicated at the bottom of each lane.

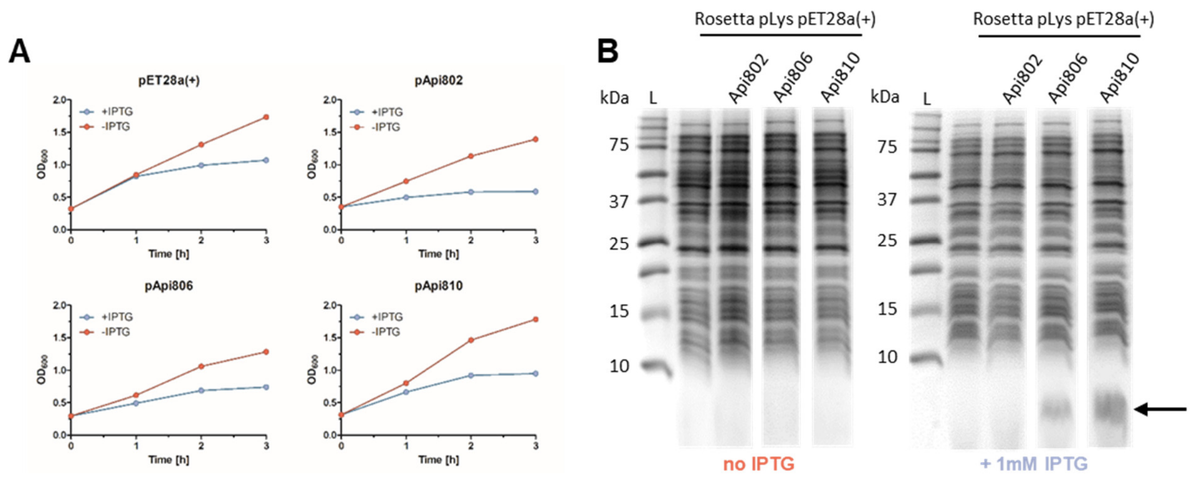

Figure S2. Bacterial growth rates and protein expression in *E. coli* Rosetta harboring an empty pET28a+ vector or pET28a+ vectors containing Api802, Api806 and Api810 sequences. **A** *E. coli* culture growth rates monitored by OD_{600} values without (red) or with (blue) inducing peptide expression by IPTG. **B** SDS-PAGE of lysates prepared from *E. coli* cells after IPTG induction. The black arrow indicates the region where Api802, Api806, and Api810 should migrate, based on free peptides.

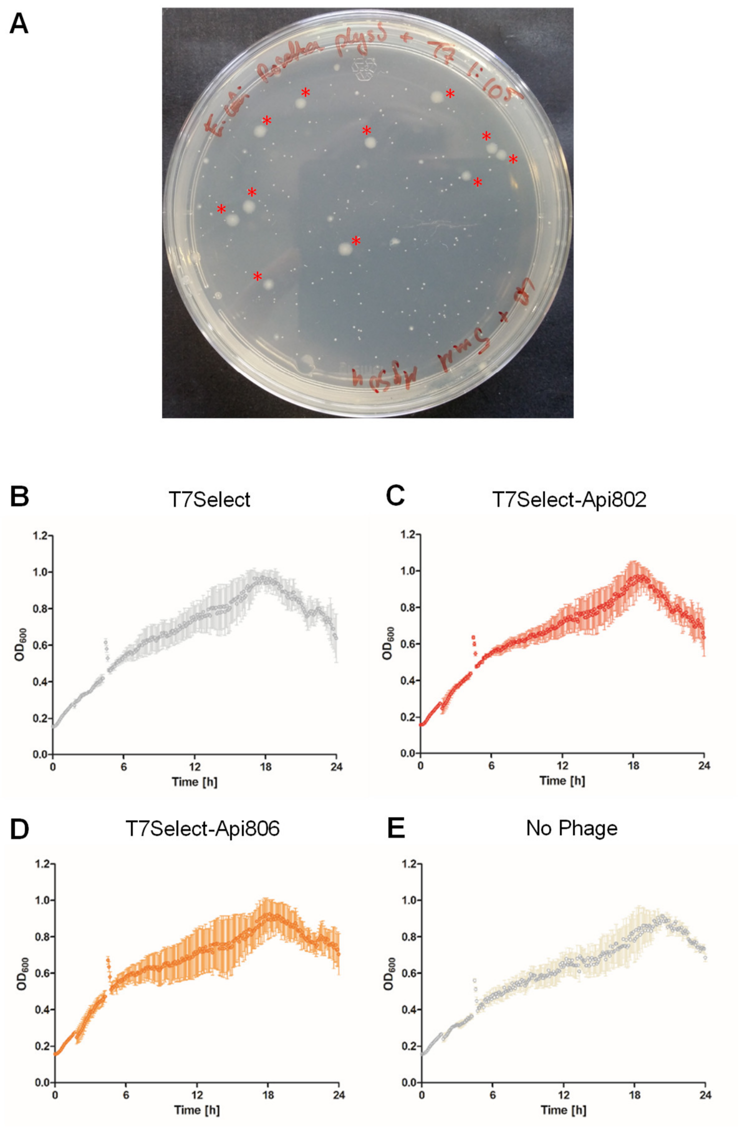

Figure S3 Isolation of phage-insensitive *E. coli* Rosetta strains. Picking of big colonies (*) of phage-insensitive strains after plaque assay with a T7Select phage titer of ~3 x 10^5 PFU/mL (**A**). Growth of the isolated T7Select phage-resistant *E. coli* Rosetta strain R2.3 infected with T7Select (**B**, gray) and engineered phages carrying Api802 (**C**, red) or Api806 (**D**, orange) inserts (MOI 0.01) or without phages (**E**, light grey). Bacterial growth was monitored by OD_{600} values recorded every 5 min for 24 h. **B-E** Experiments were done twice with six replicates. Error bars show the standard deviation of all six replicates of one representative experiment.

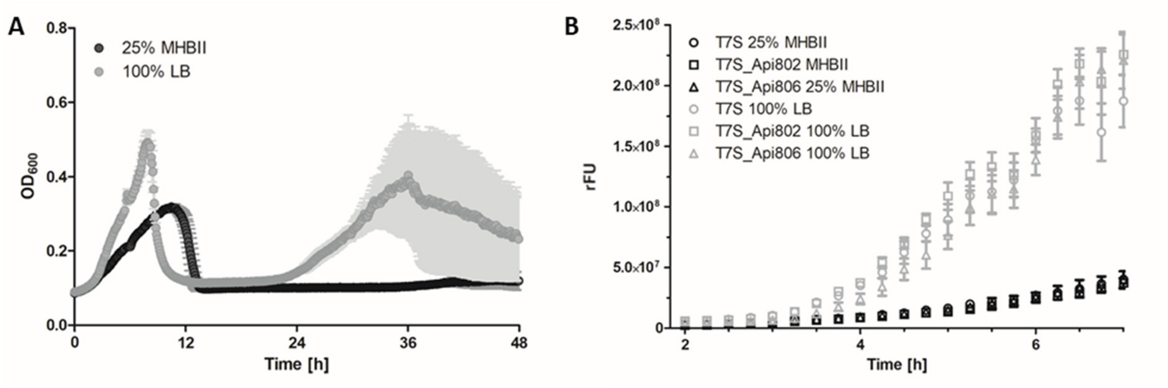

Figure S4. Lysis kinetics and protein expression are depend on the used medium. **A** Comparison of T7Select mediated lysis of *E. coli* Rosetta pLysS in 25% MHB (black) and 100% LB medium (grey). In 25% MHBII the bacterial culture does not grow as high as in 100% LB medium prior to lysis. The lysis in 25% MHBII is also delayed by roughly 2 hours. The reduced and slow growth in combination with the late lysis indicate a reduced metabolic activity of the bacteria grown in diluted MHBII. Therefore, we expected even lower expression rates of the phage integrated peptides. Additionally, there seems to be no regrowth in the minimal medium, which would have left us incapable of measuring the peptide effect on emerging phage resistant bacteria after lysis. **B** Comparison of the GFP fluorescence measured in Mixed Culture Liquid Assay in 25% MHBII and 100% LB medium. GFP expression levels in 25% MHBII are roughly 4-5 fold lower compared to those in 100% LB medium. Differences between wildtype and AMP-phages phages cannot be observed in any of the media. Experiments were done once with ten replicates. Error bars show the standard deviation of all ten replicates.
